# Inhibiting KCNMA1-AS1 promotes osteogenic differentiation of HBMSCs via miR-1303/cochlin axis

**DOI:** 10.1186/s13018-023-03538-6

**Published:** 2023-01-30

**Authors:** Yuan Lin, Hanhao Dai, Guoyu Yu, Chao Song, Jun Liu, Jie Xu

**Affiliations:** 1 Department of Orthopedics, Fujian Provincial Hospital, Fuzhou, China; 2 Shengli Clinical Medical College of Fujian Medical University, Fuzhou, China; 3 Clinical College of Orthopedics, Tianjin Medical University, Tianjin, China; 4 Department of Joints, Tianjin Hospital, Tianjin University, Tianjin, China

**Keywords:** Osteoporosis, Osteogenic differentiation, miR-1303, Cochlin, KCNMA1-AS1, Competing endogenous RNA

## Abstract

**Objective:**

Osteoporosis is a progressive systemic skeletal disorder. Multiple profiling studies have contributed to characterizing biomarkers and therapeutic targets for osteoporosis. However, due to the limitation of the platform of miRNA sequencing, only a part of miRNA can be sequenced based on one platform.

**Materials and methods:**

In this study, we performed miRNA sequencing in osteoporosis bone samples based on a novel platform Illumina Hiseq 2500. Bioinformatics analysis was performed to construct osteoporosis-related competing endogenous RNA (ceRNA) networks. Gene interference and osteogenic induction were used to examine the effect of identified ceRNA networks on osteogenic differentiation of human bone marrow-derived mesenchymal stem cells (HBMSCs).

**Results:**

miR-1303 was lowly expressed, while cochlin (COCH) and KCNMA1-AS1 were highly expressed in the osteoporosis subjects. COCH knockdown improved the osteogenic differentiation of HBMSCs. Meanwhile, COCH inhibition compensated for the suppression of osteogenic differentiation of HBMSCs by miR-1303 knockdown. Further, KCNMA1-AS1 knockdown promoted osteogenic differentiation of HBMSCs through downregulating COCH by sponging miR-1303.

**Conclusions:**

Our findings suggest that the KCNMA1-AS1/miR-1303/COCH axis is a promising biomarker and therapeutic target for osteoporosis.

**Supplementary Information:**

The online version contains supplementary material available at 10.1186/s13018-023-03538-6.

## Introduction

Osteoporosis is a progressive systemic skeletal disorder characterized by low bone density and bone destruction [1]. It is estimated that osteoporosis has affected more than 200 million populations and results in reduced quality of life and a relatively higher rate of morbidity and mortality [2]. Thereby, it is urgent to detect and intervene at the early stage of osteopenia.

Considering their aberrant expression in the pathological process of osteoporosis and their potential of being reliable biochemical indicators, increasing attention is paid to non-coding RNAs (ncRNAs), which account for more than 80% of the total human transcriptome. MicroRNAs (miRNAs) and long non-coding RNAs (lncRNAs) are two subgroups of ncRNAs with 18–25 bp and more than 200 bp in length, respectively [3], and both play crucial roles in a variety of musculoskeletal conditions [4, 5], including bone homeostasis. Inoue et al. reported miR-182 overexpression during osteoclastogenesis [6]. LncRNAs were also found dysregulated in osteoporotic mice induced by ovariectomy [7]. However, the underlying mechanism of these effects remains yet to be elucidated.

The competing endogenous RNAs (ceRNAs) hypothesis, which holds that lncRNAs can compete for binding regions of miRNAs as the molecular sponge and regulate the translation of mRNAs [8], has been verified in many diseases, including osteoporosis. Zhang et al. found that lncRNA NEAT1 and bone morphogenetic protein (BMP) 1 were dysregulated in osteoporosis and competed for miR-29b-3p as well as regulated osteogenic differentiation in bone marrow-derived mesenchymal stem cells (BMSCs) [9]. As a ceRNA of miR-214, lncRNA KCNQ1OT1 can increase the expression level of BMP2 in osteogenic BMSCs by sponging miR-214 [10]. In addition, lncRNA OGRU acts as a ceRNA to sponge miR-320-3p and protects bone mass from unloading-induced bone loss by upregulating the expression of Hoxa 10 in osteoblast [11]. Yang et al. [12] reported that competing binding of lncRNA ORLNC1, miR-296, and Pten in BMSCs regulated bone mass. These findings indicate that the overexpression and deficiency of miRNAs and lncRNAs are implicated in the initiation and development of osteoporosis. Therefore, miRNAs and lncRNAs with ceRNA activity could be biomarkers and therapeutic targets for osteoporosis. To this aim, osteoporosis-relative ceRNA networks were constructed by Zhang et al. [13] and [14] based on Gene Expression Omnibus (GEO) database. However, these two studies constructed the ceRNA networks based on the same miRNA dataset GSE62589. In addition, due to the limitation of RNA sequencing for miRNAs which depends on the platform used, the miRNAs that can be detected are incomplete. Therefore, performing RNA sequencing for miRNAs based on different platforms and screening novel miRNAs with ceRNA activity based on other osteoporosis-associated datasets may help to identify novel biomarkers and therapeutic targets for osteoporosis.

In this study, we first performed RNA sequencing for miRNA in osteoporosis subjects based on Illumina Hiseq 2500. Then, we identified differentially expressed mRNAs and lncRNAs in the GSE35958 microarray data as well as performed functional enrichment analysis. KCNMA1-AS1/miR-1303/cochlin (COCH) axis was identified as a novel biomarker and therapeutic target for osteoporosis. And the regulatory role of KCNMA1-AS1/miR-1303/COCH axis in the osteogenic differentiation of human BMSCs (HBMSCs) is experimentally validated.

## Materials and methods

### Clinical bone sample procurement

This study was approved by the Medical Ethics Committee of Fujian Provincial Hospital (No. K2019-03-034). Informed consents from the patients were obtained. The bone samples were harvested from patients who were subjected to total hip replacement operations for femoral neck fractures at Fujian Provincial Hospital from 2019 to 2020. Three female patients without osteoporosis were enrolled as the control group, while three female patients with osteoporosis were enrolled as the osteoporosis group. Bone mineral density (BMD) was measured by the double-energy X-ray absorption method. Patients with BMD ≤ − 2.5 standard deviation (SD) were diagnosed as osteoporosis. Patients with diabetes, hyperthyroidism, osteomalacia, osteopsathyrosis, rheumatoid arthritis, multiple myeloma, and bone tumor were excluded. The femoral necks were harvested during the surgeries and then stored at − 80 °C.

### RNA isolation

The samples were placed in 1 ml TRIzol (15596018, Invitrogen, USA) under liquid nitrogen. Total RNA was separated by phenol‐chloroform (P120617, Aladdin, China) extraction. Then, the RNA was treated with DNase I (18047019, Invitrogen, USA). Subsequently, a spectrophotometer (Nanodrop 1000, Thermo Scientific, USA) was used to measure the purity (OD260/OD280) of separated RNA (resuspended in 50 μl RNase-free water). Finally, a fluorimeter (Qubit 2.0, Thermo Scientific, USA) was used to quantify the accurate concentration of total RNA, while a bioanalyzer (Agilent DNA 1000 chips/Reagents, Agilent Technologies, USA) was used to assess the quality of recovered RNA to ensure that the recovered total RNA was of sufficient quality for preparing sequencing libraries.

### miRNA library preparation and RNA sequencing

miRNA library was prepared by using a Small RNA Sample Prep Kit (RS-200-0012, Illumina, USA). cDNA was converted from total RNA followed by being amplified by polymerase chain reaction (PCR). Then, the concentration of the miRNA library was quantified by using a fluorimeter (Qubit 2.0, Thermo Scientific, USA). Next, we diluted the miRNA library to 1 ng/μl. Subsequently, a bioanalyzer (Agilent DNA 1000 chips/Reagents, Agilent Technologies, USA) was used to measure the insert size of the miRNA library. And qRT-PCR was used to detect the effective concentration of the miRNA library. The effective concentration of miRNA library > 2 nM was considered satisfactory. Deep sequencing was performed on HiSeq 2500 (Illumina HiSeq 2500, Illumina, USA).

### Microarray data

The GSE74209 microarray data (five control subjects and five osteoporosis subjects), the GSE63446 microarray data (six control subjects and six osteoporosis subjects), and the GSE35958 microarray data (four control subjects and five osteoporosis subjects) were obtained from the GEO (http://www.ncbi.nlm.nih.gov/geo/).

### Acquisition of expression profiles and analysis of differentially expressed miRNAs, mRNAs, and lncRNAs

The expression profiles of mRNAs and lncRNAs were obtained from the GSE35958 profiled on GPL570. And the mRNAs and lncRNAs were mapped into the official gene symbol using the R package “biomart” [15].

Differential expression of miRNAs, mRNAs, and lncRNAs was identified by using the R package “limma” [16]. The cutoff criteria were |log2 fold change (FC)|> 0.5 and adjusted *P* < 0.05. The heatmaps of differentially expressed lncRNAs, miRNAs, and mRNAs were formed by using the R package “pheatmap” [17]. And the graphs of the expression level of differentially expressed lncRNAs, miRNAs, and mRNAs were generated by using GraphPad Prism 8 (Graph Software, San Diego, CA, USA).

### Function and pathway enrichment analysis

Functional enrichment analysis of Gene Ontology (GO) and Kyoto Encyclopedia of Genes and Genomes (KEGG) for differentially expressed mRNAs was performed by using the R package “clusterprofiler” [18]. The cutoff criteria were *P* < 0.05. The mRNAs enriched in GO terms involved in bone morphogenesis were selected, and the chord diagram was formed by R package “GOplot” [19].

### Prediction of the interaction between lncRNA and miRNA and the interaction between miRNA and mRNA

The predicted miRNA–lncRNA interaction data were collected from the LncBase V3 database (https://diana.e-ce.uth.gr/lncbasev3/interactions) and the lncRNASNP database (http://bioinfo.life.hust.edu.cn/lncRNASNP#!/). The predicted miRNA–mRNA interaction data were collected from the miRWalk 3.0 database (http://mirwalk.umm.uni-heidelberg.de/) and the TargetScan database (http://www.targetscan.org/vert_80/).

### Cell culture

HBMSCs (BMHX-C106, Cas9XTM, China) and human embryonic kidney 293T (293T) cells (TCH-C101, Cas9XTM, China) were purchased from Cas9XTM Biotechnology. HBMSCs were incubated in HBMSCs growth medium (BMHX-G101, Cas9XTM, China) at 37 °C in 5% CO_2_. 293T cells were incubated in 293T growth medium (TCH-G101, Cas9XTM, China) at 37 °C in 5% CO_2_. And cells were passaged by trypsin when they reached 70–80% confluence.

### Lentiviral transfection

Lentiviral vectors knockdown miR-1303 (si-miR-1303), COCH (si-COCH), KCNMA1-AS1 (si-KCNMA1-AS1), or negative control (si-NC) were purchased commercially (Zolgene, China). Cells were seeded into 6-well plates and allowed to grow until 50% confluent. Then, lentivirus was added to cells at a multiplicity of infection (MOI) of 50 along with 5 μg/ml polybrene. After 12 h, fresh medium was added. Next, the antibiotic selection was performed by adding 0.5 μg/ml puromycin into the medium at each medium replacement for 7 days.

### Dual-luciferase reporter assay

A Dual Luciferase Reporter Gene Assay Kit (KGAF040, KeyGEN Biotech, China) was used to validate the interaction between mRNA and miRNA. pGL3 vectors were constructed containing either wild-type (WT) or mutant (Mut) 3’-UTR region with the putative miRNA binding site of COCH mRNA or KCNMA1-AS1. The vectors were then transfected into 293T cells either with or without a miR-1303 mimic. The activities of firefly luciferase values were measured 48 h after transfection by a luminometer (SpectraMax iD5, Molecular Devices, China) and were normalized to Renilla luciferase values.

### Osteogenic differentiation

The osteoblasts were seeded in 12-well plates and incubated at 37 °C with 5% CO_2_. After 100% confluence, the medium was replaced by an osteogenic induction medium (MUBMX-90021, Cyagen, China). Western blotting and immunofluorescence staining of RUNX family transcription factor 2 (RUNX2) and osterix were performed on the 7th day of osteogenic differentiation. Western blotting of type I collagen (COL1A1) and Alizarin Red staining was performed on the 21st day of osteogenic differentiation. The staining areas of Alizarin Red were quantified by using Image J (NIH, Bethesda, USA).

### Quantitative real-time PCR (qRT-PCR)

Total RNAs from tissues were extracted by using TRIzol. For miRNA, Bulge-LoopTM miRNA qRT-PCR Starter Kit (R11067.3, RiboBio, China) was used to generate cDNA according to the manufacturer’s instructions. And Bulge-LoopTM miRNA qRT-PCR Primer (R10031.8, RiboBio, China) was used to perform qRT-RCP, and U6 small nuclear RNA was used as an internal control. For COCH and KCNMA1-AS1, First-stand cDNA Synthesis (D7168M, Beyotime, China) was used to generate cDNA according to the manufacturer’s instructions. Real-time PCR reactions were performed using a BeyoFast™ SYBR Green One-Step qRT-PCR Kit (D7268S, Beyotime, China). And glyceraldehyde-phosphate dehydrogenase (GAPDH) served as an internal control. Gene expression was quantified by using the 2 − ΔΔCt method. The primer sequences are shown as follows:

- miR-99b-5p:
- 5′-CACCCGUAGAACCGACCUUGCG-3′;
- miR-4534:
- F: 5′-GTGATTTATTTTTGTAAGTTTAGTATTTTGGGAG-3′,
- R: 5′-ACAAATACRTACCACCATACCCAA-3′;
- miR-1303:
- F, 5′-GCCGAGTTTAGAGACGGGGT-3′,
- R, 5′-CTCAACTGGTGTCGTGGA-3′;
- miR-5195-3p:
- F, 5’-GCCTGTAGGCATCATCGCCAG-3’,
- R, 5’-GATAGAGTGACGTGAAGTAG-3’;
- U6:
- F, 5′-CTCGCTTCGGCAGCACA-3′,
- R, 5′-AACGCTTCACGAATTTGCGT-3′;
- COCH:
- F, 5′-ACATCGAGGAAGCAGGCATTG-3′,
- R, 5′-TGTGACATCCTGAACCATCCC-3′;
- Retinoic acid receptor gamma (RARG):
- F, 5′-TTGAGGATGACTCCTTGCAGCCTGGTCCC-3′,
- R, 5′-GGGACCAGGCTGCAAGGAGTCATCCTCAA-3′;
- KCNMA1-AS1:
- F, 5′-TCTTTGCTCTCAGCATCGGTG-3′,
- R, 5′-CCGCAAGCCGAAGTAGAGAAG-3′;
- GAPDH:
- F, 5′-GTGGACCTGACCTGCCGTCT-3′,
- R, 5′-GGAGGAGTGGGTGTCGCTGT-3′.

#### Western blotting analysis

The cells were incubated with the lysis buffer (KGP2100, KeyGEN Biotech, China). Then we transferred the proteins onto PVDF membranes. Next, the PVDF membranes were blocked and incubated with primary antibodies against COCH (1:1000, PA5-48475, Invitrogen, USA), RUNX2 (1:2000, A11753, ABclonal, China), Osterix (1:1000, ab209484, Abcam, UK), COL1A1 (1:2000, A1352, ABclonal, China, and GAPDH (1:10,000, HRP-60004, ProteinTech, USA) at 4 °C overnight. Then, the membranes were incubated with goat anti-rabbit IgG (H + L) HRP (1:10,000, 70-GAR0072, MultiSciences, China) at room temperature (RT) for 1 h. A tanon™ high-sig ECL western blotting substrate (180-5001, Tanon, China) and automatic digital gel/chemiluminescence image analysis system (4600SF, Tanon, China) were used to visualize the immune complexes.

### Alkaline phosphatase (ALP) staining

The ALP staining was performed using an ALP stain kit according to the manufacturer’s instructions. Briefly, the cells were fixed with 4% paraformaldehyde for 15 min at RT. Then, the cells were incubated with ALP incubation solution at 37 °C for 12 h. After washing with running water for 2 min, the cells were incubated with the Co solution at 37 °C for 5 min. Then, the cells were rinsed 3 times and incubated with a vulcanizing working solution for 2 min at RT.

### Immunofluorescence staining

The cells were fixed with 4% paraformaldehyde for 15 min at RT. Then, cells were blocked with quickblock blocking buffer for immune staining (P0260, Beyotime, China) for 15 min at RT, followed by incubation with primary antibody against RUNX2 (1:200, A2851, ABclonal, China), or osterix (1:1000, ab209484, Abcam, UK) at 4 °C overnight and labeled with Alexa Fluor594-preabsorbed goat anti-rabbit IgG (ab150084, Abcam, 1:500, UK) for 2 h at RT. Next, the nucleus was stained with DAPI.

### Statistical analysis

We performed all statistical analyses and generated the graphs using GraphPad Prism 9 (Graph Software, San Diego, CA, USA). Shapiro–Wilk test for normal distribution and Bartlett’s test for homogeneity of variance were performed. Then, statistical significance was determined by Student’s *t* tests, one-way analysis of variance (ANOVA), or two-way ANOVA with Tukey tests for multiple comparisons. Pearson correlation analysis was performed. We presented all data as mean ± standard deviation and considered values of *P* < 0.05 significant.

## Results

### Differential expression of miR-1303 in osteoporosis subjects

**Fig. 1:**
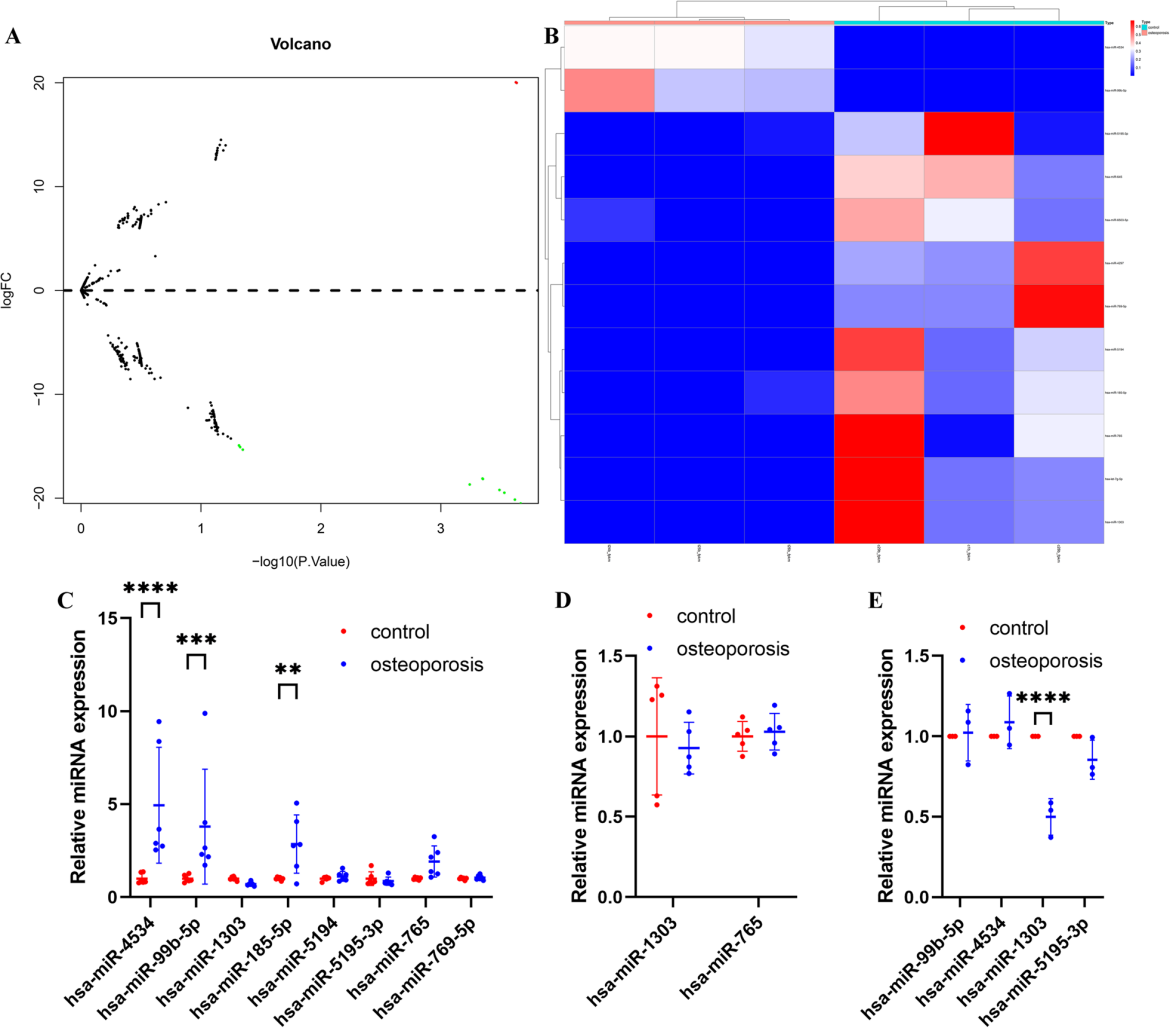
miR-1303 is downregulated in osteoporosis subjects. **A** Volcano plots of differentially expressed miRNAs. **B** Hierarchical clustering heatmap of differentially expressed miRNAs (red: high expression, white: medium expression, and blue: low expression). **C** Verification of the expression pattern of the differentially expressed miRNAs in the GSE74209. **D** Verification of the expression pattern of the differentially expressed miRNAs in the GSE63446. **E** The expression of identified miRNAs in the osteoporotic subjects and the control subjects is determined by qRT-PCR. **C–E** Values are shown as mean ± SD. **P < 0.01, ***P < 0.001, ****P < 0.0001, two-way ANOVA

To identify differentially expressed miRNAs in osteoporotic patients from our RNA sequencing results, a comparative analysis was performed. With a |log2FC| cutoff criteria > 0.5 and *P* value < 0.05, we identified 12 miRNAs as differentially expressed miRNAs in osteoporotic subjects compared with healthy control subjects (Fig. 1A) (Additional file 1: Table S1). Among them, there were 2 upregulated miRNAs and 10 downregulated miRNAs (Fig. 1A). Subsequently, we performed hierarchical clustering of these differentially expressed miRNAs to visualize the expression patterns. The heatmaps showed that the expression level of these differentially expressed miRNAs was able to distinguish osteoporotic subjects from healthy control subjects (red: high expression, white: medium expression, and blue: low expression) (Fig. 1B).

To further verify the expression profiles of the above 12 miRNAs, we applied these miRNAs to 2 independent osteoporosis microarray datasets of miRNAs (GSE74209 and GSE63446). We found that in the GSE74209 and the GSE63446, only miR-4534, miR-99b-5p, miR-1303, and miR-5195-3p showed a trend consistent with our sequencing results (Fig. 1C, D). Thus, we next performed qRT-PCR to verify the expression levels of these 4 miRNAs. qRT-PCR indicated that only miR-1303 was decreased significantly in osteoporotic subjects (Fig. 1E).

### COCH knockdown improves osteogenic differentiation of HBMSCs

**Fig. 2:**
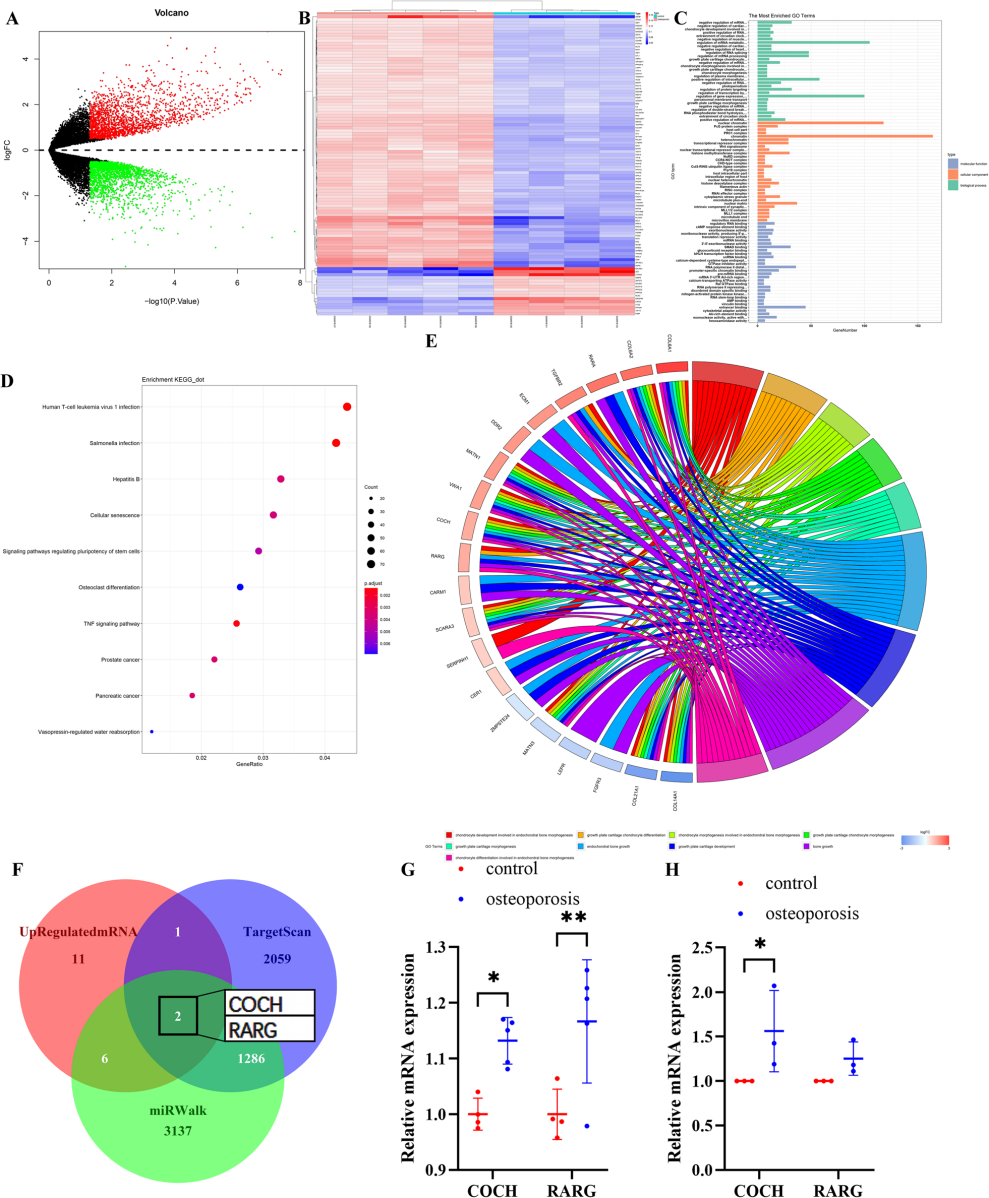
COCH is identified as a promising downstream mRNA of miR-1303. **A** Volcano plots of differentially expressed mRNAs. **B** Hierarchical clustering heatmap of differentially expressed mRNAs (red: high expression, white: medium expression, and blue: low expression). **C** Enrichment of top 30 GO terms. **D** Top 10 enriched KEGG pathways. The node color changes from red to blue in descending order according to the adjusted p values. The size of the node depends on the number of counts. **E** Chord diagram of the differentially expressed mRNAs according to the results of the GO pathway involved in bone morphogenesis. The box color changes from red to blue in descending order according to the logFC of differentially expressed mRNAs. **F** Venn diagram of the osteoporosis-related upregulated mRNAs and the predicted mRNAs with the ability to interact with miR-1303. **G** Verification of the expression pattern of COCH and RARG in the GSE35958. **H** The expression of COCH and RARG in the osteoporotic subjects and the control subjects is determined by qRT-PCR. **G**, **H** Values are shown as mean ± SD. *P < 0.05, **P < 0.01, two-way ANOVA

Subsequently, we aimed to screen osteoporosis-related mRNA which was mediated by has-miR-1303. Comparative analysis was performed to identify differentially expressed mRNAs in the GSE35958. 4178 differentially expressed mRNAs were identified, including 2052 upregulated mRNAs and 2126 downregulated mRNAs (Fig. 2A) (Additional file 2: Table S2). Hierarchical clustering showed that the expression level of these mRNAs could distinguish osteoporotic subjects from healthy control subjects (red: high expression, white: medium expression, and blue: low expression) (Fig. 2B).

Then, we performed GO functional enrichment analysis for the differentially expressed mRNAs. GO functional enrichment analysis indicated that the differentially expressed mRNAs were mainly involved in the negative regulation of mRNA metabolic process, negative regulation of cardiac muscle hypertrophy, and chondrocyte development involved in endochondral bone morphogenesis in the biological process group (Fig. 2C). And these differentially expressed mRNAs were mainly enriched in nuclear chromatin, PcG protein complex, and host cell part in the cellular component group (Fig. 2C). In addition, these differentially expressed mRNAs were mainly involved in regulatory RNA binding, cAMP response element binding, and exoribonuclease activity in the molecular function group (Fig. 2C). We also performed KEGG pathway analysis for these differentially expressed mRNAs. The data showed that these differentially expressed mRNAs were markedly involved in human T-cell leukemia virus 1 infection, salmonella infection, and hepatitis B (Fig. 2D).

To further visualize differentially expressed mRNAs enriched in the terms related to osteoporosis, a chord diagram was used (Fig. 2E). A total of 20 mRNAs were enriched in 9 GO terms related to bone formation and reabsorption among 78 GO analysis results in the biological metabolic process. Next, by using the TargetScan database and miRWalk database, two upregulated mRNAs (COCH and RARG) with the ability to interact with miR-1303 were screened (Fig. 2F). Both COCH and RARG were elevated about 1.1–1.2 times in the GSE35958 (Fig. 2G). However, by performing qRT-PCR, we found that COCH was increased significantly while RARG showed a mild elevation without statistic difference in the osteoporosis subjects compared to the control subjects (Fig. 2H).

**Fig. 3:**
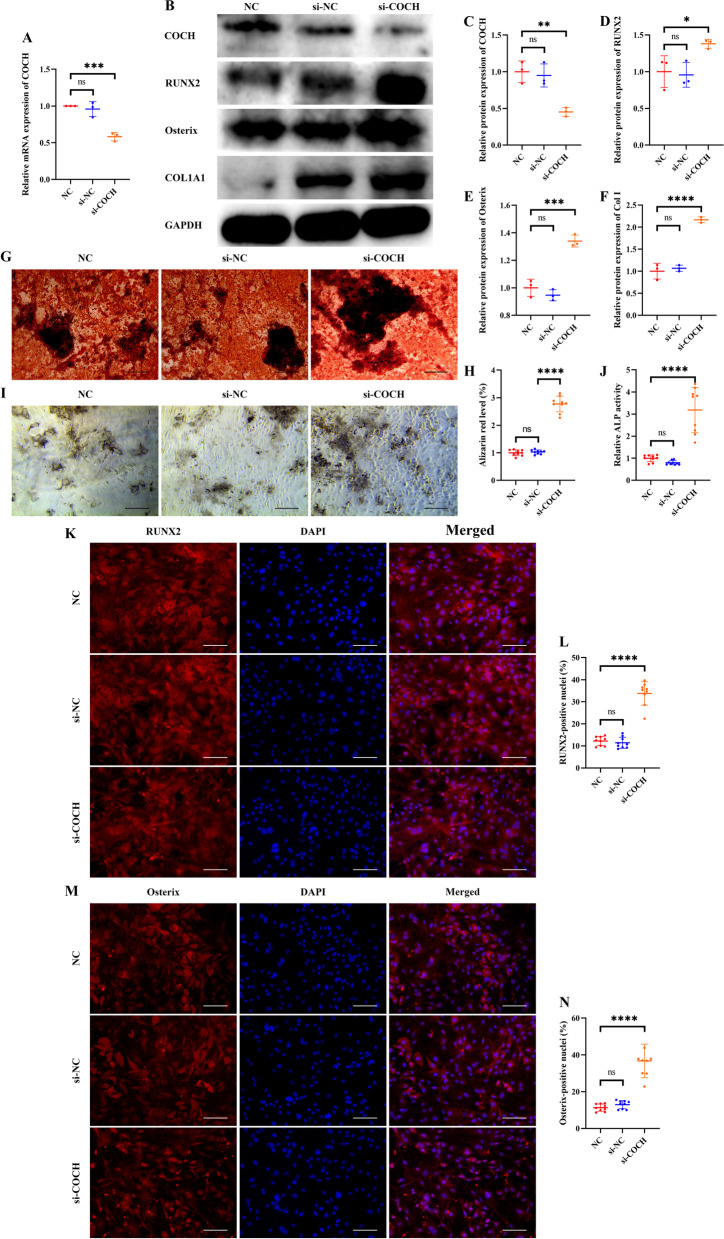
COCH knockdown promotes osteogenic differentiation of HBMSCs. **A** Transfection efficacy after COCH interference is determined by qRT-PCR. **B–F** Western blotting (**B**) and quantitative analysis of COCH (**C**), RUNX2 (**D**), Osterix (**E**), and COL1A1 (**F**) in HBMSCs treated with si-NC and si-COCH. **G**, **H** Staining of calcium deposition by Alizarin Red (**G**) and quantitative analysis of staining areas (**H**) in HBMSCs treated with si-NC and si-COCH. Scale bars, 200 μm. **I**, **J** Staining of ALP (**I**) and quantitative analysis of staining areas (**J**) in HBMSCs treated with si-NC and si-COCH. Scale bars, 200 μm. **K**, **L** Immunofluorescence staining of RUNX2 (**K**) and quantitative analysis of RUNX2-positive nuclei (**L**) in HBMSCs treated with si-NC and si-COCH. Scale bars, 100 μm. **M**, **N** Immunofluorescence staining of Osterix (**M**) and quantitative analysis of RUNX2-positive nuclei (**N**) in HBMSCs treated with si-NC and si-COCH. Scale bars, 100 μm. **A**, **C–F**, **H**, **J**, **l**, **N** Values are shown as mean ± SD. *P < 0.05, **P < 0.01, ***P < 0.001, ****P < 0.0001, one-way ANOVA

To investigate the role of COCH in regulating osteogenic differentiation, HBMSCs were treated with either si-COCH or si-NC. qRT-PCR and western blotting showed that COCH levels were downregulated by about 40–50% by si-COCH treatment (Fig. 3A–C). Protein levels of RUNX2, Osterix, and COL1A1, three markers of osteogenic differentiation, were upregulated by COCH knockdown (Fig. 3b–f). And more mineral deposition and higher ALP activity were found in COCH knockdown HBMSCs than that in the NC group and si-NC group (Fig. 3G–J). Also, COCH knockdown improved the nuclear translocation of RUNX2 and osterix (Fig. 3k–n).

### miR-1303 mediates osteogenic differentiation of HBMSCs via sponging COCH

**Fig. 4:**
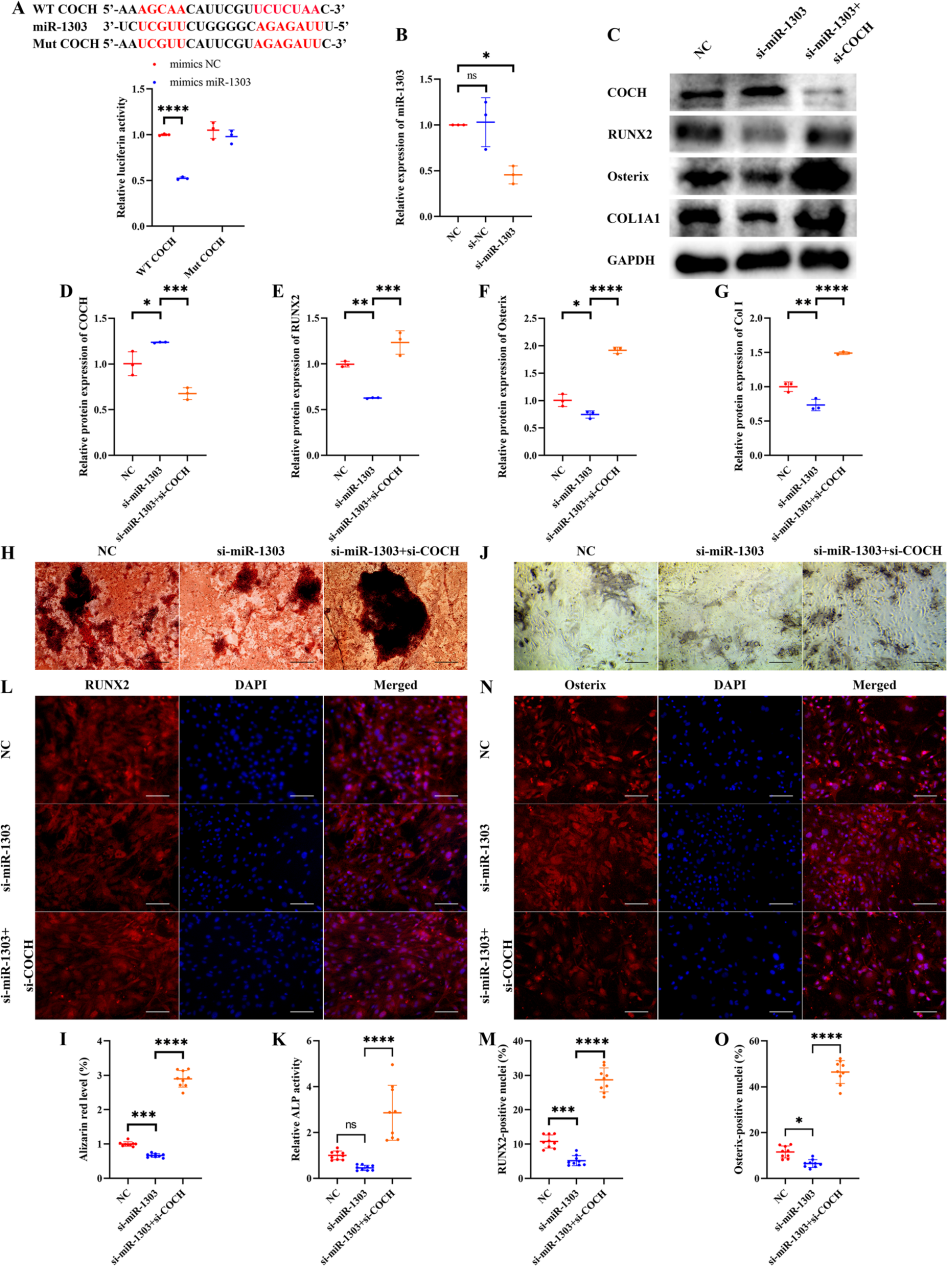
miR-1303 mediates osteogenic differentiation of HBMSCs by sponging COCH. **A** The predicted binding site between the miR-1303 and COCH, and the luciferase activity of the WT COCH and Mut COCH in 293T cells treated with mimics miR-1303 or mimics NC. **B** Transfection efficacy after miR-1303 interference is determined by qRT-PCR. **C–G** Western blotting (**C**) and quantitative analysis of COCH (**D**), RUNX2 (**E**), Osterix (**F**), and COL1A1 (**G**) in HBMSCs treated with si-miR-1303 and si-miR-1303 plus si-COCH. **H**, **I** Staining of calcium deposition by Alizarin Red (**H**) and quantitative analysis of staining areas (**I**) in HBMSCs treated with si-miR-1303 and si-miR-1303 plus si-COCH. Scale bars, 200 μm. **J**, **K** Staining of ALP (**J**) and quantitative analysis of staining areas (**K**) in HBMSCs treated with si-miR-1303 and si-miR-1303 plus si-COCH. Scale bars, 200 μm. **L**, **M** Immunofluorescence staining of RUNX2 (**L**) and quantitative analysis of RUNX2-positive nuclei (**M**) in HBMSCs treated with si-miR-1303 and si-miR-1303 plus si-COCH. Scale bars, 100 μm. **N**, **O** Immunofluorescence staining of Osterix (**N**) and quantitative analysis of RUNX2-positive nuclei (**O**) in HBMSCs treated with si-miR-1303 and si-miR-1303 plus si-COCH. Scale bars, 100 μm. **A** Values are shown as mean ± SD. ****P < 0.0001, two-way ANOVA. **B**, **D–G**, **I**, **K**, **M**, **O** Values are shown as mean ± SD. *P < 0.05, **P < 0.01, ***P < 0.001, ****P < 0.0001, one-way ANOVA

The binding site of miR-1303 and COCH was predicted by using the TargetScan database. Luciferase results showed that miR-1303 mimics observably downregulated the luciferase activity of the WT COCH, while no effects on the Mut COCH (Fig. 4A), suggesting that miR-1303 could directly sponge COCH. Then, the si-miR-1303 was used to knock down miR-1303. The level of miR-1303 declined by about 60% in the si-miR-1303 group compared to the NC group and the si-NC group (Fig. 4B). Western blotting showed that the protein level of COCH in HBMSCs was visibly increased after si-miR-1303 treatment (Fig. 4C, D). And the expression levels of RUNX2, Osterix, and COL1A1 were significantly decreased in the miR-1303 knockdown group, while COCH knockdown could effectively reverse the negative effects of miR-1303 knockdown on the protein levels of RUNX2, Osterix, and COL1A1 (Fig. 4C–G). Furthermore, we consistently found that miR-1303 knockdown reduced mineral deposition, ALP activity, and the nuclear translocation of RUNX2 and osterix, which could be attenuated by COCH knockdown (Fig. 4H–O).

### KCNMA1-AS1 promotes osteogenic differentiation of HBMSCs through miR-1303/COCH axis

**Fig. 5:**
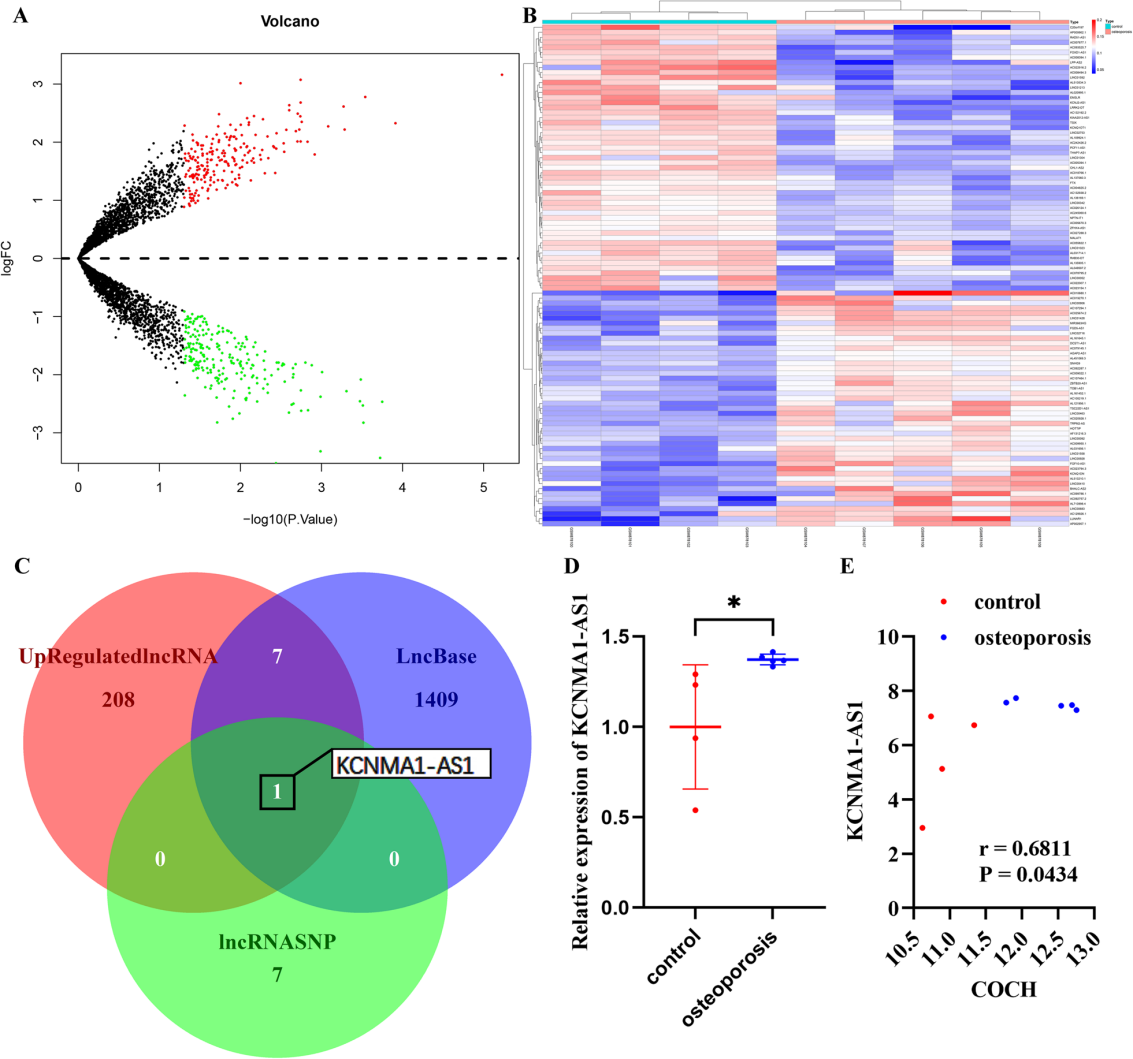
KCNMA1-AS1 is screened as a candidate for upstream lncRNA of mir-1303. **A** Volcano plots of differentially expressed lncRNAs. **B** Hierarchical clustering heatmap of differentially expressed lncRNAs (red: high expression, white: medium expression, and blue: low expression). **C** Venn diagram of the upregulated lncRNAs and the predicted lncRNAs with the ability to interact with miR-1303. **D** Verification of the expression pattern of KCNMA1-AS1 in the GSE35958. **E** The Pearson correlation of the expression value of KCNMA1-AS1 and COCH in the GSE35958. **D** Values are shown as mean ± SD. *P < 0.05, Student’s t test.

Comparative analysis was performed to identify differentially expressed lncRNAs in the GSE35958. 437 lncRNAs were identified as differentially expressed lncRNAs. Among them, 216 lncRNAs were upregulated and 221 lncRNAs were downregulated (Fig. 5A) (Additional file 3: Table S3). As shown in hierarchical clustering, the expression level of these lncRNAs could distinguish osteoporotic subjects from healthy control subjects (red: high expression, white: medium expression, and blue: low expression) (Fig. 5B). Subsequently, we screened one upregulated lncRNA, KCNMA1-AS1, which could interact with miR-1303 by using LncBase database and lncRNASNP database (Fig. 5C). KCNMA1-AS1 was increased about 1.4 times in the osteoporosis subjects (Fig. 5D). In addition, the Pearson correlation between the expression value of KCNMA1-AS1 and COCH in the GSE35958 was 0.6811 (Fig. 5E).

**Fig. 6:**
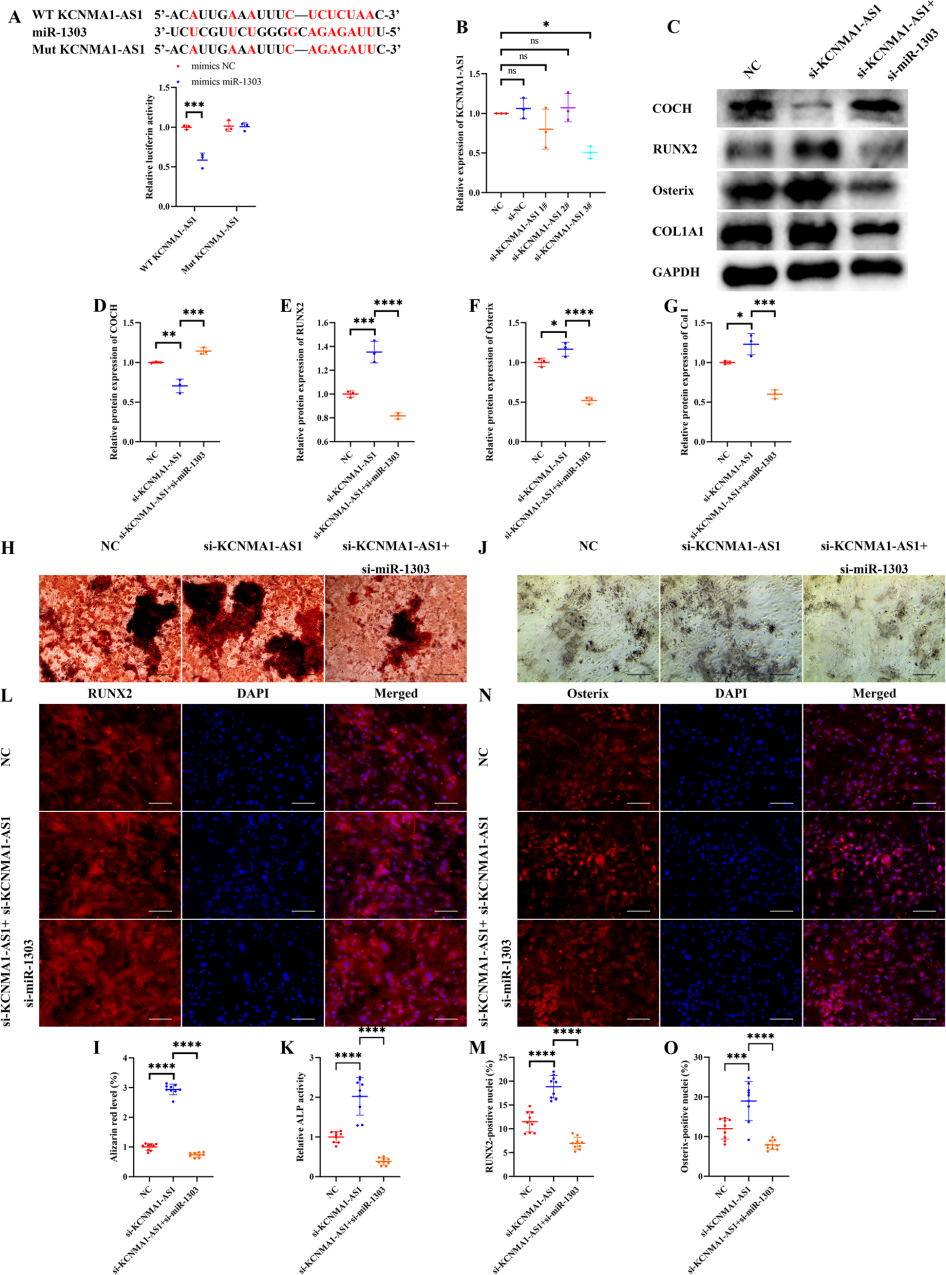
KCNMA1-AS1 promotes osteogenic differentiation of HBMSCs through miR-1303/COCH axis. **A** The predicted binding site between the miR-1303 and KCNMA1-AS1, and the luciferase activity of the WT KCNMA1-AS1 and Mut KCNMA1-AS1 in 293T cells treated with mimics miR-1303 or mimics NC. **B** Transfection efficacy after KCNMA1-AS1 interference is determined by qRT-PCR. **C–G** Western blotting (**C**) and quantitative analysis of COCH (**D**), RUNX2 (**E**), Osterix (**F**), and COL1A1 (**G**) in HBMSCs treated with si-KCNMA1-AS1 and si-KCNMA1-AS1 plus si-miR-1303. **H**, **I** Staining of calcium deposition by Alizarin Red (**H**) and quantitative analysis of staining areas (**I**) in HBMSCs treated with si-KCNMA1-AS1 and si-KCNMA1-AS1 plus si-miR-1303. Scale bars, 200 μm. **J**, **K** Staining of ALP (**J**) and quantitative analysis of staining areas (**K**) in HBMSCs treated with si-miR-1303 and si-miR-1303 plus si-COCH. Scale bars, 200 μm. **L**, **M** Immunofluorescence staining of RUNX2 (**l**) and quantitative analysis of RUNX2-positive nuclei (**M**) in HBMSCs treated with si-miR-1303 and si-miR-1303 plus si-COCH. Scale bars, 100 μm. **N**, **O** Immunofluorescence staining of Osterix (**N**) and quantitative analysis of RUNX2-positive nuclei (**O**) in HBMSCs treated with si-miR-1303 and si-miR-1303 plus si-COCH. Scale bars, 100 μm. **A** Values are shown as mean ± SD. ***P < 0.001, two-way ANOVA. **B**, **D–G**, **I**, **K**, **M**, **O** Values are shown as mean ± SD. *P < 0.05, **P < 0.01, ***P < 0.001, ****P < 0.0001, one-way ANOVA

The lncRNASNP database was used to predict the binding site of miR-1303 and KCNMA1-AS1. We found that miR-1303 mimics significantly inhibited the luciferase activity of the WT KCNMA1-AS1 but not the Mut KCNMA1-AS1 (Fig. 6A). KCNMA1-AS1 knockdown HBMSCs were constructed by using si-KCNMA1-AS1. qRT-PCR showed that only si-KCNMA1-AS1 3# could effectively reduce the expression of KCNMA1-AS1 (Fig. 6B). To investigate whether KCNMA1-AS1 regulated osteogenic differentiation through miR-1303/COCH axis, si-miR-1303 were transfected in KCNMA1-AS1 knock-downed HBMSCs. KCNMA1-AS1 knockdown inhibited the protein expression of COCH, while miR-1303 restraint rescued the expression of COCH (Fig. 6C, D). The protein levels of RUNX2, Osterix, and COL1A1 were all increased after KCNMA1-AS1 knockdown, which was counteracted by miR-1303 knockdown (Fig. 6C, E–G). Consistent results were observed in the Alizarin Red staining and ALP staining that KCNMA1-AS1 knockdown improved the mineral deposition and ALP activity of HBMSCs, while miR-1303 knockdown eliminated beneficial effects of KCNMA1-AS1 knockdown on the mineral deposition and ALP activity of HBMSCs (Fig. 6H–K). In addition, the nuclear translocation level of RUNX2 and osterix in HBMSCs was increased after KCNMA1-AS1 knockdown. But the beneficial effects of KCNMA1-AS1 knockdown on the nuclear translocation of RUNX2 and osterix were significantly declined when miR-1303 was downregulated (Fig. 6L–O).

## Discussion

With the balance of osteoblastic bone formation and osteoclastic bone absorption destroyed, bone loss and bone destruction occur, which is the pathological basis of osteoporosis. Osteoporosis patients receive high clinical attention due to the increased risk of fracture [20]. Mechanically, osteoporosis is partly caused by the dysregulation of bone metabolism in BMSCs and osteoblasts, thus failing to induce enough osteoblasts to prevent bone resorption by osteoclasts [21]. Therefore, the changing molecules during osteogenic differentiation of BMSCs are valuable diagnosis and treatment targets for osteoporosis [22]. There are already many indexes, such as bone mineral density (BMD), alkaline phosphatase (ALP), and vitamin D, serving as diagnostic markers for osteoporosis in clinical [23]. As we mentioned in the introduction part, the ncRNAs, including miRNAs and lncRNAs, have been reported being able to play the same role in early stage of osteopenia [13, 14, 24, 25].

In the present study, we first performed RNA sequencing for miRNAs based on Illumina Hiseq 2500, whose application in osteoporosis, to our best knowledge, has not been reported yet. We found 12 dysregulated miRNAs in the osteoporosis subjects. However, in the GSE74209 and GSE63446, only 4 miRNAs (miR-4534, miR-99b-5p, miR-1303, and miR-5195-3p) matched our sequencing results. Then, qRT-PCR is performed to verify the expression levels of these miRNAs in the osteoporosis subjects. And qRT-PCR indicated that only the change in miR-1303 was statistically significant. Though it has been reported that miR-1303 can mediate cellular proliferation in several cancers [26–31], miR-1303 has not been investigated in osteoporosis.

We further screened the downstream mRNAs of miR-1303 based on the ceRNA hypothesis. At present, some studies have already reported ceRNA networks in BMSCs in osteoporosis by analyzing microarray datasets [13, 14]. For example, Wang et al. found that intervening OGRU/miR-320-3p/Hoxa 10 signaling axis alleviated unloading-induced osteoporosis [11]. And Yang et al. [12] reported that lncRNA ORLNC1 regulated bone mass through miR-296/Pten axis. Also, there already are some studies that analyzed DE genes in the microarray dataset GSE35958 [32–35]. In the present study, the GSE35958 was repurposed. And we provided 2 osteoporosis-related mRNAs with ceRNA activity (COCH, RARG) as promising candidate downstream mRNAs of miR-1303. Ivanovska et al. reported that inhibiting RARG drove osteogenesis [36]. Surprisingly, qRT-PCR showed that the elevation of RARG in the osteoporosis subjects was not significant, while the mRNA level of COCH was increased significantly. Though COCH has not been reported in osteoporosis, Zhang et al. found that COCH improved self-renew but suppressed the differential potential of embryonic stem cells [37]. So, we deduce that COCH may play a negative role in the osteogenesis of HBMSCs. It has been reported that small interfering RNAs (siRNAs) have been widely used as a tool for knockdown gene expression in the studies of several orthopedic diseases [38, 39]. So, we used siRNAs to knockdown COCH, miR-1303, and KCNMA1-AS1 in this study. And our hypothesis was verified experimentally that COCH knockdown promoted the expression of osteogenic markers and mineral deposition of HBMSCs. In addition, we found that miR-1303 knockdown positively regulated COCH expression directly, and subsequently inhibited osteogenic differentiation of HBMSCs.

Finally, we aim to screen the upstream lncRNAs of miR-1303. Only one lncRNA, KCNMA1-AS1, was found to be increased in the osteoporosis subjects and equipped with the ability to interact with miR-1303. Moreover, KCNMA1-AS1 and COCH got Pearson correlation coefficients > 0.5. Unfortunately, KCNMA1-AS1 has not been studied in osteoporosis. And Ma et al. found that KCNMA1-AS1 could resist apoptosis of epithelial ovarian cancer cells by targeting Caspase-9 and Caspase-3 [40]. Dual-luciferase reporter assay demonstrated the direct interaction between KCNMA1-AS1 and miR-1303. Furthermore, we found that KCNMA1-AS1 knockdown accelerated the osteogenic differentiation of HBMSCs via the miR-1303/COCH axis.

The present study still has some limitations. First, we only explored the molecular mechanism of the KCNMA1-AS1/miR-1303/COCH axis regulating the osteogenic differentiation of HBMSCs in vitro. Further animal experiments are needed to certify the feasibility of the KCNMA1-AS1/miR-1303/COCH axis as a potential therapeutic target for osteoporosis. Second, we only performed miRNA sequencing in 6 bone samples from female subjects. More samples should be collected and sequenced from both female and male subjects.

In conclusion, we showed that miR-1303 was downregulated, while KCNMA1-AS1 and COCH were upregulated in osteoporosis subjects. KCNMA1-AS1 mediated osteogenic differentiation of HBMSCs via miR-1303/COCH axis. Summarily, our findings suggest that the KCNMA1-AS1/miR-1303/COCH axis is a promising biomarker and therapeutic target for the diagnosis and treatment of osteoporosis.

## Supplementary Information

**Additional file 1: Table S1.** Differentially expressed miRNAs between control subjects and osteoporosis subjects.

**Additional file 2: Table S2.** Differentially expressed mRNAs between control subjects and osteoporosis subjects in the GSE35958.

**Additional file 3: Table S3.** Differentially expressed lncRNAs between control subjects and osteoporosis subjects in the GSE35958.

## Data Availability

The datasets generated during and/or analyzed during the current study are available from the corresponding author upon reasonable request.
